# Points from Sessional Addresses

**Published:** 1909-10-16

**Authors:** 


### Points from Sessional Addresses.

### THE WISDOM OF THE WISE.

**Pioneers on Pioneers.**

We are one and all too apt to step in and enjoy what has been won for us by the labours of our predecessors as if it has been bestowed, like manna from Heaven, as the bounteous gift of Providence, without a thought of the years of courage and self-sacrifice which have been necessary to attain it. The vision, the compelling necessity, the accompanying self-sacrifice come to one man in ten million; he moves forward, removes difficulties, shows the way, and then the mass follows and enjoys the fruits of his labours and probably hardly give a thought to him who has put these advantages within reach. Just as a healthy person does not think about his health, so those who enjoy what others have won seldom think how those benefits are obtained. Still, there is a certain ungraciousness and ingratitude in accepting all that has been won by the labour of others without any sort of acknowledgment. There is only one really valid excuse for this attitude, and that is ignorance.—*Mrs. Fawcett at The Royal Free.*

**The Clinical Value of Anatomy and Physiology.**

It is in the surgical wards chiefly that anatomical knowledge becomes specially useful, as it enables us to locate the organ in which the disease is situated and to avoid during subsequent surgical treatment structures of vital importance; while during the medical work the interpretation of symptoms, the daily examinations of body fluids, etc., required by every clinical clerk, are simply the application of principles already learnt in the physiological laboratory. Finally, in the examination of the patients themselves, the condition of the pulse, blood, blood pressure, heart sounds, reflexes, special senses, etc., a knowledge of physiology, together with a little common sense and manual dexterity, are the only assets really required.—*Dr. Goodall at The Middlesex.*

**Vivisection.**

It is instructive, in view of the modern outcry against the experimental method, to read Jones's *Apologia,* bearing in mind that his experiments were performed before the days of anæsthetics and painless operations. "I regret," he wrote, "the necessity of obtaining this important knowledge by the sacrifice of brutes, but when we remember the incessant scourge of war which has followed man through all the ages of his history, not to mention the consequences of accident and disease, it is not too much to assert that thousands might have been, and may still be, saved by the knowledge of these subjects which can only be directly obtained by experiments on brutes, indirectly and very slowly by observations on the injured arteries of men, and even this cannot be made until he has fallen a sacrifice to the want of assistance or to the imperfect knowledge of the surgeon." Every practical surgeon will bear testimony that as the result of Jones's experiments tens of thousands of lives have been saved. As future practitioners of medicine you will have to form an opinion on the justifiability of experiments upon living animals for the purposes of scientific research, and if you are satisfied that these experiments are justifiable, with proper safeguards, you will be called upon to defend the practice and to justify your opinion. To do this intelligently it is necessary not only to be acquainted with the arguments for the defence, but also to make yourselves masters of the arguments on the other side. It is not enough to ascribe the opposition of the public to ignorance or fanaticism. There may be either or both, but objection can be only overcome and acquiescence gained by enlightening and educating the public mind.—*Sir John Tweedy at University College.*

**Essentials of Success.**

A knowledge of patients themselves and of individual peculiarities is essential for the successful practice of your profession. The importance of a psychic factor is well recognised in ordinary physiological processes; in digestion, for instance, with a psychic stimulus (appetite) we digest four times as much food in a given time as without it; while as regarded disease, the psychic factor is, from the patient's point of view, the worst in every case. Surely it is a factor which must be recognised and treated just as much as the actual pathological condition. The danger of this belief lies in the fact that you may exaggerate its importance, and so over-treat it, to the neglect of the actual physical factor. Ignore the psychic factor and your treatment may, in the case of a sensitive patient, amount to actual brutality. Over-treat the psychic factor, on the other hand, and you yourself become a quack and run the risk of making your patient a chronic neurasthenic.—*Dr. Goodall at The Middlesex.*

**Hobbies and Holidays.**

I would strongly urge upon you the importance of the "holiday habit" and the following of some pursuit to take you as medical men away from your work. Driving a motor-car has often added new interest to the round of a medical man, and has likewise cultivated the habit of rapid observation and immediate action. It is also most valuable to try and see what is being done elsewhere. Go abroad and see for yourselves what other men are doing at other hospitals and universities. It is a common thing in Germany for a student to take at least one of his years at another university.—*Dr. George Murray at Manchester.*

**Mistaken Theory.**

Another educational theory is the view that you ought to be always amassing a knowledge of facts not for present but for future use. Persons brought up in this spirit not only lose all the present interest in the vitality of their work, but live for the rest of their lives in a continual state of expectation or preparation, and are investing their intellectual resources in promise instead of in performance.—*Dr. Miers at Mary's.*

**The Importance of Research.**

The great *desideratum* of our day is adequate endowment of biological and pathological research as the surest and most humane way of discovering the nature and cause of disease and its best prevention. Clinical observation only deals with disease when and as it manifests itself in man and animals; it throws but little light upon the causes of disease or upon the intimate processes which constitute the pathological state. Supremely useful as hospitals are for the purpose of treating the sick by means of the best skill and knowledge of the time, and as schools are for the training of successive generations of medical practitioners for the service of the community, it is nevertheless true that most of the capital discoveries of scientific medicine have been made outside hospitals and very often apart from clinical observation altogether.—*Sir John Tweedy at University College.*

